# Molecular classification based on apomorphic amino acids (Arthropoda, Hexapoda): Integrative taxonomy in the era of phylogenomics

**DOI:** 10.1038/srep28308

**Published:** 2016-06-17

**Authors:** Hao-Yang Wu, Yan-Hui Wang, Qiang Xie, Yun-Ling Ke, Wen-Jun Bu

**Affiliations:** 1 Institute of Entomology, College of Life Sciences, Nankai University, Tianjin 300071, China; 2 College of Computer and Control Engineering, Nankai University, 38 Tongyan Road, Haihe Education Park, Jinnan District, Tianjin 300350, China; 3 Guangdong Entomological Institute, Guangzhou 510260, China

## Abstract

With the great development of sequencing technologies and systematic methods, our understanding of evolutionary relationships at deeper levels within the tree of life has greatly improved over the last decade. However, the current taxonomic methodology is insufficient to describe the growing levels of diversity in both a standardised and general way due to the limitations of using only morphological traits to describe clades. Herein, we propose the idea of a molecular classification based on hierarchical and discrete amino acid characters. Clades are classified based on the results of phylogenetic analyses and described using amino acids with group specificity in phylograms. Practices based on the recently published phylogenomic datasets of insects together with 15 *de novo* sequenced transcriptomes in this study demonstrate that such a methodology can accommodate various higher ranks of taxonomy. Such an approach has the advantage of describing organisms in a standard and discrete way within a phylogenetic framework, thereby facilitating the recognition of clades from the view of the whole lineage, as indicated by PhyloCode. By combining identification keys and phylogenies, the molecular classification based on hierarchical and discrete characters may greatly boost the progress of integrative taxonomy.

Taxonomy is the science of classifying, describing, identifying, and naming. With interdisciplinary endeavours becoming increasingly common in biological research, an accurate and stable classification system is prerequisite due to its role as one of the cornerstones for the integration of multiple fields. The precise description of a clade can provide both information from the past and inspiration for the future. Over the past two centuries, developments in the life sciences have involved the concomitant adjustment of the hierarchical and binomial classification systems developed by Carolus Linnaeus. Among these, perhaps the most important ontological change of the original system is that biological classifications should now be phylogenetic, *i.e.*, each group that is recognised in a classification should be monophyletic^1^,^2^,^3^,^4^. With the purpose of defining and naming clades in the tree of life with more explicit reference to a phylogeny, the idea of phylogenetic nomenclature has been developed that uses the phylogenetic definitions of taxa and a rank-free classification system^1^,^5^,^6^,^7^,^8^. In the context of phylogenetic nomenclature, the information provided by rank-signifying ending is limited. In addition, supraspecific names are not always explicitly associated with clades under the rank-based codes, resulting in ambiguous definitions and an impediment in the clear communication and efficient storage and retrieval of biological information. Therefore, some rank-free phylogenetic classifications^1^,^5^,^6^,^7^,^8^ have been suggested to replace rank-based ones. Instead of a total negation, a series of modifications of the classic system with hierarchy have been proposed by the opponents of phylogenetic nomenclature. For example, Platnick advocated an extension of the standard rank-based classification through the implementation of rank-based definitions to the names of clades at all hierarchical levels^9^, and Ward suggested maintaining a ranked phylogenetic taxonomy, at least for groups in relatively recent and species-rich branches of the tree of life^10^.

Setting the controversy on the ontological issues of biological entities aside, both the proponents and opponents of the phylogenetic nomenclature have agreed that the current rank-based classification system cannot meet the need for describing the growing levels of evolutionary divergences revealed by the great advancement of phylogenetics. Indeed, from the perspective on standardisation and efficiency, such a challenge also necessitates the development of taxonomic epistemology and methodology. For example, the traditional classification systems for both animals and plants are mainly based on morphological characteristics. Therefore, for practical considerations, clades that can be named should be both strongly supported as monophyletic groups in phylogenetic analyses and have distinctive phenotypic features that allow them to be distinguished from related taxa^10^, *i.e.*, correctly classified and precisely described. However, not all of the clades that we would like to study have such definitive diagnostic morphological traits. Furthermore, due to the continuous variation of many morphological traits, for many clades, it seems inevitable to employ morphological definitions that involve unique and conditional combinations of traits rather than clear and unequivocal synapomorphies^10^. In addition, because there are only a few homologous morphological traits at higher category levels due to the difficulty in establishing general ground-plans, it is hard to apply a classification system that allows for meaningful comparisons in different groups of organisms. In all, the way thus far adopted for descriptions and diagnoses of clades restrict the further development of taxonomy.

With the great advance of high-throughput sequencing technology in the last decade, it has become possible to rapidly and economically acquire large amounts of genome or transcriptome sequences. The steadily declining sequencing costs make it no longer inhibitory to analyse transcriptomes or even whole genomes, which can boost the development of a molecular-based taxonomic classification system. Currently, the major approaches that use huge molecular data sets to describe clades are based on similarity. For example, Marakeby *et al*.^11^ proposed an exclusively genome-based classification and naming system, in which the proposed organism codes are assigned based on measured similarity. However, a similarity-based classification system may not accurately reflect evolutionary relationships, *i.e.*, a code may be assigned to a paraphyletic or polyphyletic clade rather than a monophyletic one, which violates the broad consensus of current biological classification. Meanwhile, with the discoveries of many lineage-specific nucleotide/amino acid residues in comparative studies, a series of character-based approaches have also been proposed, *e.g.*, ribosomal multilocus sequence typing, which assigns bacteria to genetic lineages that have identical alleles at certain genomic loci^12^. Nevertheless, the application of these approaches is restricted to certain groups, and the relationships between different sequence types are uninformative due to the lack of information on hierarchy.

Herein, we propose a hierarchical character-based molecular classification that uses group-specific traits of multilocus genes as the description and diagnoses of clades. In this classification frame, only characters with definite diagnosability can be candidates for the description of clades, *i.e*., the amino acids or nucleotides that differ among organisms will not be treated as equal. Each clade in a lineage is given a unique code that is derived from a mining of group-specific apomorphies, *i.e.*, a trait that is found in some or all terminal groups of a clade and is inherited from a common ancestor^13^. A strategy based on the criterion of parsimony is introduced to detect apomorphies in a dataset. The codes are then arranged in a queue based on the taxonomic hierarchy and finally generate the complete diagnostic description of a terminal taxon (species) in the tree of life. The data from a recent published phylogenomic study about the evolution of hexapods^14^ and from a comparative genomic study of Anophelinae mosquitoes (Diptera: Culicidae)^15^ were chosen to generate two datasets for this study based on two reasons. On the one hand, insects are among the most diverse organisms on the planet, representing approximately half of all known living organisms^16^. On the other hand, several insect clades had been seen as “problematic” in the phylogenetic studies based on single or a few gene markers (*e.g.*, Polyneoptera), which makes it ideal for testing the feasibility of our system. Thousands of apomorphies were found under a strict filter criterion, covering nearly all of the nodes in the original studies. The results of the phylogenetic reconstructions based on such sites showed abundant phylogenetic signals. Two classification systems were constructed according to the category levels between the superclass and the order and between the subfamily and the species complex. Our study provides a cladistic approach to reanalyse the molecular sequence data of a phylogenomic study and shows the potential of a synthesis in systematic biology, whereby phylogenomics, molecular classification, and PhyloCode may be integrated.

## Results

### Apomorphy mining and the subset optimisation

After apomorphy mining with filter criteria based on various consistency index (CI) values, we generated several sub-datasets. For the dataset of Hexapoda, a total of 7,939, 8,008, and 11,241 apomorphies were identified to generate sub-datasets 1A, 1B, and 1C, respectively. For the Anophelinae dataset, a total of 422, 422, and 464 apomorphies were identified to generate sub-datasets 2A, 2B, and 2C, respectively. The phylogenetic trees inferred from the six sub-datasets are shown in Figs S1 and S2. For the phylogenetic inference on Hexapoda, only the tree based on sub-dataset 1B retrieved the same topology as the original. Meanwhile, for the phylogenetic reconstruction of Anophelinae, both the topology and support values obtained from sub-datasets 2A, 2B and 2C were acceptable. As a result, considering both phylogenetic reappearance and the informativeness for apomorphy mining, sub-datasets 1B and 2C were used for subsequent analyses.

### Building the code-system of Hexapoda using molecular apomorphies

**Figure 1:**
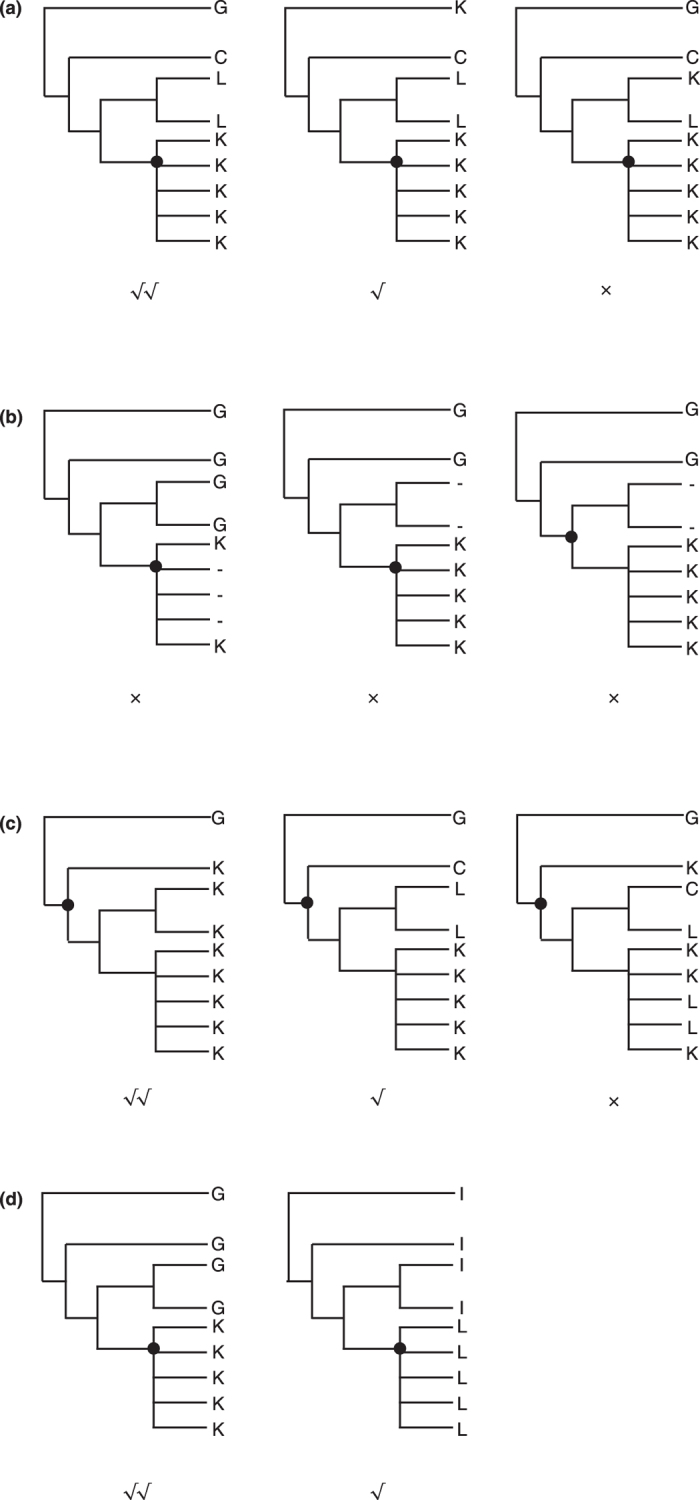
Summary of the strategy in selecting apomorphies. The black dot indicates the group of organism as the goal clade. The cross indicates that such a scenario should be refused, while the tick indicates that such a scenario can be accepted. Double ticks indicate an acceptance with high priority. (**a**) Preference of apomorphies based on the extent of overlapping. (**b**) Preference of apomorphies based on the data coverage in a site. (**c**) Preference of apomorphies based on the extent of uniqueness. (**d**) Preference of apomorphies based on the rarity of amino acids substitution.

**Figure 2:**
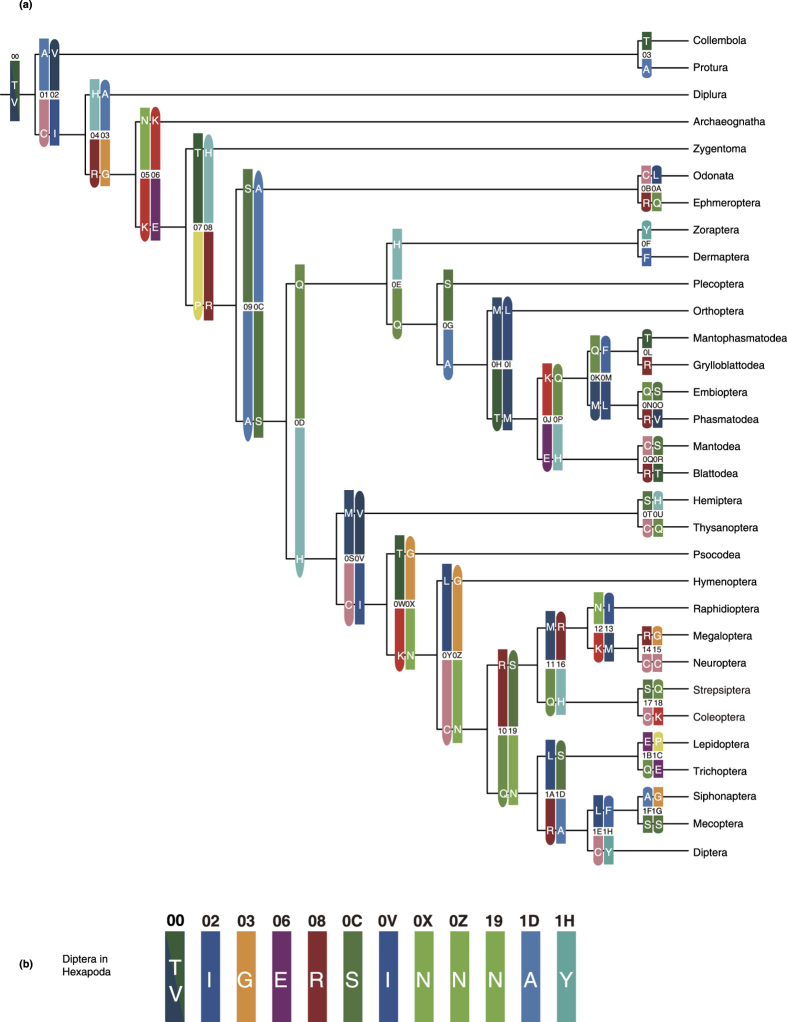
Molecular descriptions of clades in Hexapoda based on apomorphic amino acids. The apomorphies of amino acids are coloured based on the respective biochemical attributes. States shown in rounded rectangles indicate plesiomorphic states, while states shown in rectangles indicate apomorphic states. The diagonal indicates a binary apomorphic state. (**a**) Tree-like descriptions for clades in Hexapoda. (**b**) Combined description for Diptera.

**Figure 3:**
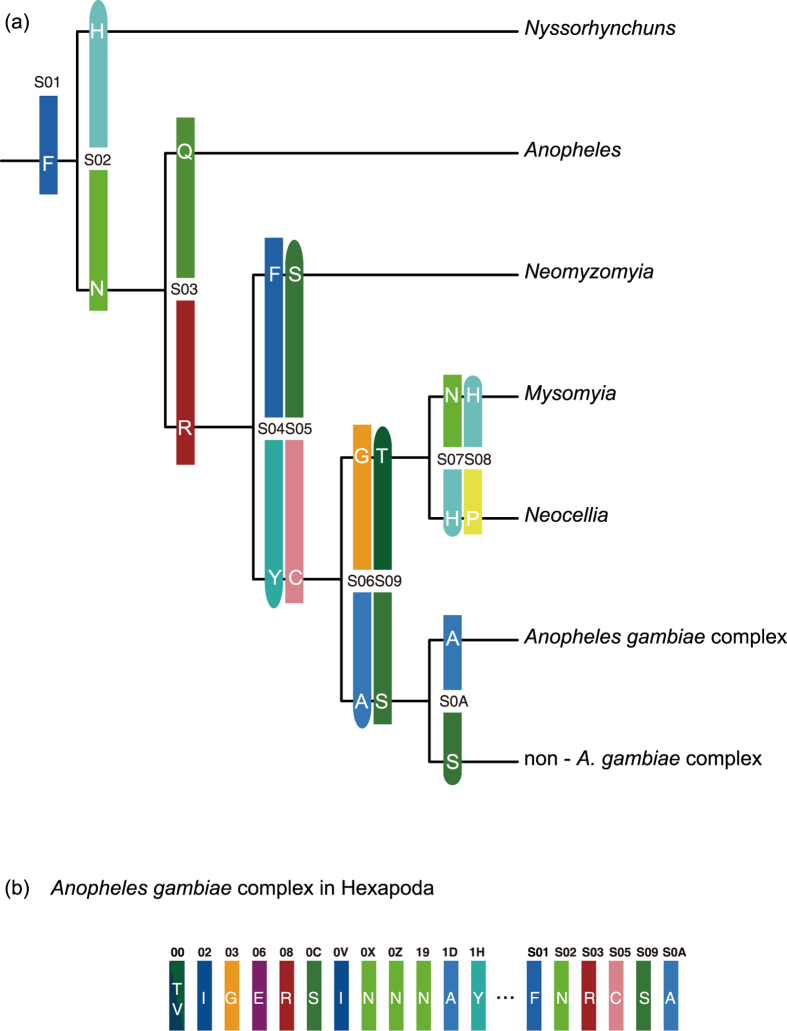
Molecular descriptions of clades in Anophelinae based on apomorphic amino acids. The apomorphies of amino acids are coloured based on the respective biochemical attributes. States shown as a rounded rectangle indicate plesiomorphic states, while states shown as a rectangle indicate apomorphic states. The diagonal indicates a binary apomorphic state. (**a**) Tree-like descriptions for clades in Anophelinae. (**b**) Combined description for *Anopheles gambiae* complex.

**Figure 4:**
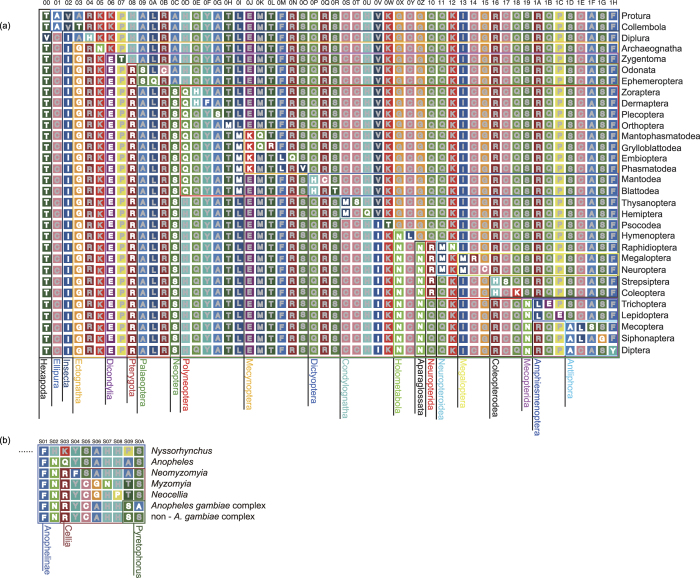
Sequential descriptions of clades based on apomorphic amino acids shown in a two-dimensional table. (**a**) Sequential descriptions of clades in Hexapoda. (**b**) Sequential descriptions of clades in Anophelinae. The number above each column is a numerical symbol. Apomorphies that are confirmed to be unique by comparing all of the organisms in the dataset are shown in white text. Non-apomorphic characters are shown in grey text. Each description for a lineage consists of two parts. The substantial parts for identification comprise apomorphic codes that are arranged following a strict hierarchical order (corresponding to the bars of discrete symbol). While the subordinate and trivial parts comprise the non-apomorphic characters, which only plays a structurally appurtenant role and contain no information for description and diagnoses (corresponding to the additional space of discrete symbologies). It should be noted that the minor variations in non-apomorphic characters are not shown for simplification, albeit the proportion of which are very small.

From the potential apomorphies in sub-datasets 1B and 2C, a series of sites were selected based on a group of optimal criteria (Fig. 1) to construct two classification systems according to category levels between the class and order in Hexapoda and between the subfamily and species complex in Anophelinae. The results are shown in Figs 2a and 3a, which correspond to the topologies revealed by phylogenomics. The sequential arrangements of codes in two-dimensional tables are shown in Fig. 4. Each code assigned to an internal node in a tree contains two types of information: the state of the corresponding element and the position where the apomorphies are located. The positional information is shown in the form of sequential IDs to facilitate presentation, the annotations of which are shown in Fig. S3. A complete informative diagnostic description of the organism is composed of codes assigned to each node in its lineage from root to leaf, corresponding to the substantial part of the sequential ones, thus exhibiting a hierarchical structure. Notably, only the codes that refer to apomorphic states are actually informative for the whole description of organisms, which are meant to ensure the independence of codes. For example, the whole apomorphy-based description of Diptera and the *Anopheles gambiae* complex in Hexapoda can be shown using the sequence |T/V|IGERSINNNAY (ID: 00, 02, 03, 06, 08, 0C, 0V, 0X, 0Z, 19, 1D, and 1H, Fig. 2b) and |T/V|IGERSINNNAY…FNRCSA (ID: 00, 02, 03, 06, 08, 0C, 0V, 0X, 0Z, 19, 1D, 1H, …, S01,S02,S03,S05,S09, and S0A, Fig. 3b), respectively. Such structure of information is similar to the rules for naming species and infraspecific taxa utilised in the latest version of PhyloCode^17^. In other words, the completely informative description of a clade can be further decomposed into two parts: a diagnostic code for a given clade and a hierarchical prefix composed of a sequential arrangement of codes assigned to the clades in which the given clade is nested.

### Query test based on unknown transcriptomes

We categorised the results of the query as follows. (1) Positive, when the queried transcriptome is assigned to a right terminal node; (2) False-positive, when the queried transcriptome is assigned to a wrong terminal node; (3) False-negative, when the queried transcriptome cannot be assigned to any terminal node in the test database due to missing states; and (4) Negative, when the queried transcriptome cannot be assigned to any terminal node in the test database due to possible variations or sequencing errors. The results of the identification are shown in Table 1 and Table S1. Despite the historical controversy on monophyly, all polyneopterans and non-polyneopterans were correctly assigned to the respective group according to the codes. Among the 51 tested transcriptomes (Table S2), no false-positive or negative results were found. Due to “missing” states at the terminal nodes, three queries retrieved false-negative results. Although many of the internal nodes had “missing” states, 48 of the 51 queries obtained positive results. Approximately 60% of these “missing” states can be attributed to missing sites of amino acids in the queried transcriptome (Table S1), which shows a semi-random distribution among transcriptomes of different sizes (Fig. S4A). The proportion of missing genes drops greatly as the size of transcriptome increases and reaches approximately zero when the size of transcriptome is over 90,000 contigs (Fig. S4B). The proportion of nodes with “missing” states first decreases steadily with an increase in the size of the transcriptome sequencing assembly but reaches a relatively stationary phase over 30,000 contigs (Fig. S4C).

## Discussion

We propose that the results of our study can greatly benefit molecular apomorphy-based classification. First, the diagnostic molecular codes with mutual independence allow a novel and concise molecular approach for taxonomists to define and describe clades via a series of apomorphic amino acids. For example, along the lineage from the root of Hexapoda to Diptera, the clade of Insecta including Diplura and Ectognatha can be described as the clade originating from the ancestor species possessing apomorphy 829I as inherited from 829V on the clathrin heavy chain, the clade of Ectognatha can be described as the clade originating from the ancestor species possessing apomorphy 961G as inherited from 961A on nuclear hormone receptor FTZ-F1, and so on, until the clade of Diptera, which can be described as the clade originating from the ancestor species possessing apomorphy 692H as inherited from 692F on ubiquitin carboxyl-terminal hydrolase (Figs 2b, 4a and S3). This coding can be further extended along the lineages within Diptera, taking the position of the *Anopheles gambiae* complex in Diptera-Anophelinae as an example (Figs 3b, 4b and S3). Such an approach can be especially useful for the category levels at which uniquely diagnostic morphological variations are occasionally rare and could hardly be used to distinguish closely related taxa.

In addition, the application of a hierarchical prefix in the description could benefit the classification from the aspect of hierarchy, in which the information regarding nesting and mutual exclusivity can be definitely provided. For example, the prefix of Diptera is |T/V|IGERSINNNA (ID: 00, 02, 03, 06, 08, 0C, 0V, 0X, 0Z, 19, and 1D); therefore, Diptera must be nested in the clades corresponding to codes in the prefix, *e.g.*, Insecta (02-I), Pterygota (08-R), and Holometabola (0X-N), but can never be nested in clades such as Polyneoptera (0D-Q). Clades are much closer if they share more similar prefixes and are judged to be the closest or sister groups if the prefixes are the same but the diagnostic codes are different. With this structure, our approach can utilise an explicit phylogenetic definition by which the clades are fully defined and described under a phylogenetic framework and show explicit references to a particular phylogenetic hypothesis, thus coinciding with the principles of PhyloCode.

It may be suggested that there are similarities between the proposed molecular apomorphy-based classification and DNA barcoding, which is a technique of specimen classification that serves an important role in assessing and describing biological diversity^18^,^19^,^20^,^21^,^22^,^23^ by using a DNA sequence from some gene (*e.g.*, cytochrome c oxidase subunit I (*COI*)) as a species-specific barcode^24^,^25^,^26^,^27^. As complete clade descriptions in our approach can be interpreted as a series of diagnostic codes along the lineages, our approach may also act as a sort of identification key and a diagnostic “barcode”. Although either approach can implement standardisation, practicability, and generality in rapid identification, the criterion of the similarity-based barcoding methods^28^,^29^ that is used to distinguish one species from others is the use of some categorised thresholds to describe gaps in genetic distances. Moreover, similarity-based strategies also restrict current DNA barcoding to a leaves-only processing that only provides one level of resolution and does not focus on precise information regarding the relationships among barcodes. Indeed, current DNA barcoding methods cannot describe a clade higher than the species level through explicit reference to phylogeny, although this is not the main question that they are designed to answer.

In contrast, the criterion applied here for distinguishing one clade from the others is qualitative rather than quantitative. A series of molecular apomorphies are used as clade identification tags that are unique for certain groups of organisms and completely distinct among different groups. Apomorphies at the amino acid level are mainly used to ease the ambiguity resulting from molecular homoplasy. As opposed to the mere assemblage of mutually exclusive characters used in diagnostic barcoding^30^,^31^, after apomorphy mining and filtering under a set of criteria, the apomorphies of various nodes should be arranged according to the rank of the node in the lineage from root to leaf, by which the hierarchical prefix and the diagnostic code of a clade can be given and combined in a particular order. In this sense, the whole description of clades in this classification system is highly hierarchical, thus facilitating phylogenetic descriptions and diagnoses of clades that we would like to study at various category levels. In this study, the concrete establishment of classification systems in Hexapoda and Anophelinae has shown the feasibility of hierarchical molecular apomorphy-based classification in multiple category levels. Additionally, at the root end, some apomorphic nucleotides/bases have been shown to exist and demonstrate the existence of group specific molecular attributes in bacteria, archaea, and eukaryotes^32^; meanwhile, at the leaf end, apomorphic amino acids/nucleotides have been successfully discovered in several species which could hardly be distinguished when using morphological characters^33^,^34^. Therefore, benefiting from the unambiguous attribute of apomorphy, a hierarchical molecular apomorphy-based classification system has the ability to put forward a general criterion without sensitivity resulting from various genetic distances among all living organisms on Earth.

**Figure 5:**
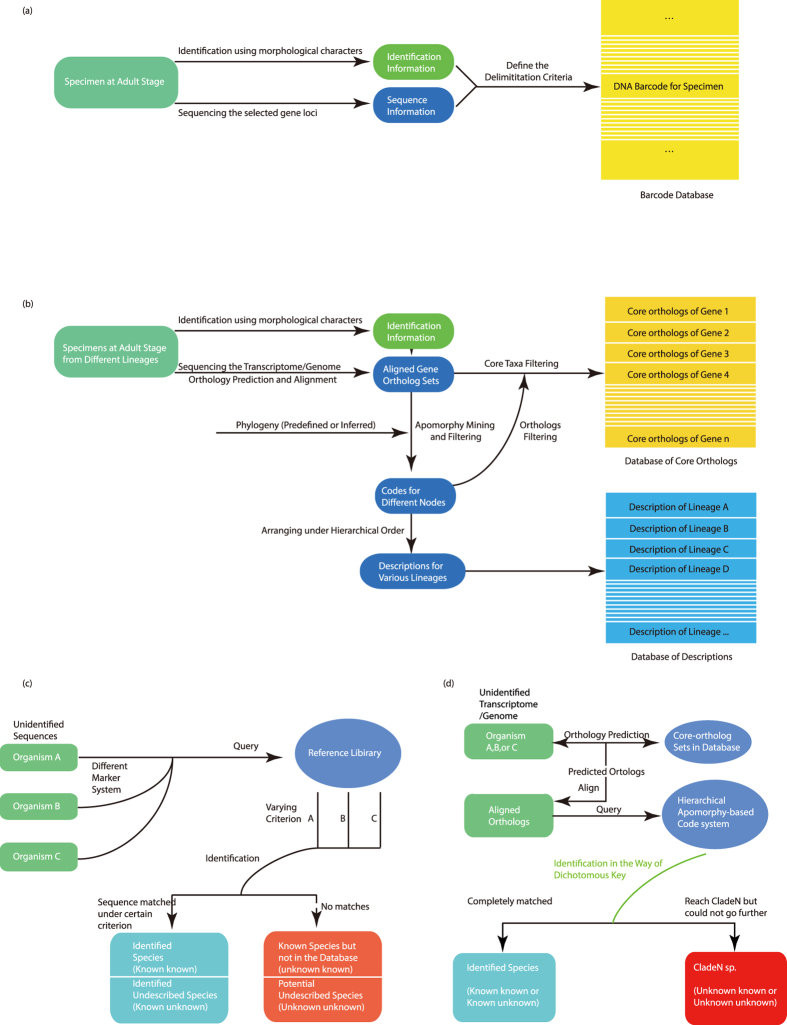
Comparison of DNA barcoding methods and the hierarchical molecular apomorphy-based classification system. Complete definition of known known, known unknown, unknown known, unknown unknown can be found in the study produced by Collins and Cruickshank^59^. (**a**) General workflow of database construction in the previous barcoding methods. (**b**) General workflow of database construction in the hierarchical molecular apomorphy-based system. (**c**) General workflow of identification in the previous barcoding methods. (**d**) General workflow of identification in the hierarchical molecular apomorphy-based classification system.

The general procedures for database construction are also different between DNA barcoding methods and the hierarchical molecular apomorphy-based classification, as shown in Fig. 5a,b. In DNA barcoding methods, the sequences of gene markers are directly deposited in the database and linked with the identification information. While in the hierarchical apomorphy-based classification, two related sub-databases should be formed simultaneously. One database contains sets of core-orthologs of each homologous gene, in which the information of apomorphic amino acids is imbedded, and the other contains descriptions, *i.e.*, sequences of codes for organisms based on discrete and apomorphic amino acids. The sub-database of core-orthologs should be updated regularly according to orthology annotation databases, such as KEGG^35^, OrthoDB^36^, OMA^37^, while the sub-database of descriptions can follow a similar way that joins published data into a fully versioned and dynamic framework^38^,^39^.

It should be noticed that the molecular apomorphy-based classification does not abandon the existing monolocus data used in molecular identification. On the contrary, compiled homologous sequences should be explored as much as possible to discover the molecular apomorphic sites, no matter whether they are short sequences or genome or transcriptome data, and no matter whether they are amino acids or nucleotides. Therefore, such inclusiveness offers an optimal utilisation of the existing sequence data for the apomorphy-based barcoding system, thus leading to standard and efficient molecular identifications in a post-genomic era. Simultaneously, the approach for molecular classification proposed here does not imply a replacement of the existing biological classification system. In fact, the apomorphy-based molecular classification should be seen as an epistemological and methodological complement and extension that could be compatible with classification systems with different ontological declarations.

Several challenges may occur during the application of a hierarchical apomorphy-based classification system. First, as shown in the query test results, missing genes and missing amino acid sites in sequenced transcriptomes will affect the efficiency and accuracy of this approach to some extent, thereby causing ambiguities in descriptions and identifications. Such challenge could hopefully be overcome in two ways in the future. As shown in Fig. S4B, the proportion of missing genes resulting from the incompleteness of the transcriptome is greatly reduced as the size of the transcriptome increases. That indicates that the problem of missing genes can be effectively solved with increases in sequencing throughput. While for the proportion of missing amino acid sites resulted from fragmented sequencing, such cases can be hopefully solved by increasing the length of reads and by increasing the completeness of assembly in the progressive high-throughput sequencing techniques^40^,^41^,^42^. Thus, the proportion of false-negative results can be reduced correspondingly.

Because the apomorphy-based system relies highly on tree topology, such a system may encounter problems if a phylogenetic ground plan has not been well established for the group under consideration. In the phylogenomic era, the currently recognised challenges include non-random distributed missing data, great rate heterogeneity, and serious incomplete lineage sorting (ILS), among others. According to a comprehensive survey using simulated and empirical big data^43^, several gene-tree-based coalescent (ASTRAL, MP-EST) and supertree (MRP) methods consistently recovered the true species tree as the number of genes increased to 1,000 even in the presence of 70% non-random missing data either in sampled taxa or in genes with high ILS and rate heterogeneity. In other words, the reliability of the reference phylogenomic trees used for molecular apomorphy-based coding can be checked by such methods and have good opportunities to be convincing with the continuously increasing –omics-based data currently and in the future.

It may be argued whether all of the qualified nodes should be encoded in a fully resolved tree when considering the convenience of taxonomic practices. Although a complete set of codes may appear to be excessive, especially compared to traditional keys and the morphological characters used in them, fully encoded clade descriptions in fact provide other conveniences. Because the storage of codes and the procedure of decoding can both be accomplished computationally, restrictions in the length of descriptions and diagnoses are indeed relaxed. In addition, similar to other methods for molecular identification, the apomorphy-based encoding can be relaxed from the restrictions of sexual dimorphism and developmental stages and the professionalism requirement for taxonomic practitioners, thus simplifying the workflow of taxonomic practices. Furthermore, because both the description and identification of a certain clade follow a tree-climbing procedure on a strictly evaluated phylogenetic tree, the dichotomous-key-like presentation of successive codes can facilitate identification, evaluation and comparisons among closely related organisms in a fully phylogenetic way. In this sense, a complete set of codes can finally achieve an accurate and strictly phylogenetic description of biological organisms. Therefore, we propose that full encoding should be encouraged rather than simply translating traditional levels or ranks into codes that are often arbitrary and may result in subsequent controversy.

It should be noted that only one apomorphy per clade was used as code in the designed database in this study for the convenience of illustration. In fact, such a scheme is expandable in a real classification system. Because rare mutants may occur at even the most conservative sites in some individuals of any species (*i.e.*, diagnostic exceptions where case subtaxa deviate from the otherwise diagnostic identity of a given state), the redundancy of multiple codes for the same clade in identification keys will be necessary when using apomorphic amino acids. The strategy of adopting multiple codes rather than one for the description and identification of a certain clade can be viewed as a supraspecific extension of the“near-minimal” set of SNPs (single nucleotide polymorphism), which are commonly used in species-level rapid identification^44^. Such a strategy may be especially important in tackling some “problematic” taxonomic groups or organisms that have experienced recent speciation. To increase the available amount of qualified apomorphies, the coverage of sites in the sequence matrix used for apomorphy mining should be as high as possible. Nevertheless, such a redundancy of apomorphies does not mean a conditional combination of traits. In fact, each apomorphy in codes with redundancy is independent from the others. The strategy of multiple encoding is only to avoid the error resulting from minor and practical constraints. On the other hand, together with the development of sequencing technologies, the mining of apomorphic amino acids can be improved by reducing missing data. Although the extent of the reference genome coverage remains biased in that there is a dearth of non-vertebrate genomes throughout the tree of life^45^, hundreds of genomes and more than hundreds of thousands of transcriptomes in eukaryotes have been sequenced. Furthermore, the development of third-generation sequencing can greatly boost the process of genome sequencing with broader taxon sampling. The eliminated need for excessive reagents and the harnessing of the processivity of DNA polymerase in third-generation sequencing allow an increase in the integrity of throughput and a decrease in the time and cost of sequencing^42^. Moreover, with the gradual accomplishment of genome annotation and orthology prediction in distantly related taxa, the number of genes that can be used for apomorphy mining will be increased accordingly. Therefore, using redundant apomorphies as identification tags for one clade is both necessary and feasible, and we propose that the permanent code for an organism should have more than one column site for each clade.

Furthermore, the challenge resulting from the high specialisation and the non-generalisation of the marker system can also be relieved or even overcome with advances in sequencing technology. In contrast to the early age of DNA barcoding using Sanger sequencing, it has become realistic in the era of high-throughput sequencing to generate a large amount of molecular data from different loci simultaneously, efficiently, and economically. The explosively increasing amount of genome and transcriptome data may even permit apomorphy mining in almost all of the extant organisms in the future. As a result, molecular classification studies have been largely freed from the restrictions of sequencing and the number of markers used. Moreover, it is realistic to compare markers from thousands of available gene sequences based on the existence of apomorphic amino acids for a certain clade, which can lead to the progressive optimisation of the marker system. Therefore, with an even more rapid accumulation of genome and transcriptome data in the future, the hierarchical apomorphy-based classification system can achieve standardisation and, thus, the gradual fixation of the marker system.

The broadness and depth of genomic and transcriptomic data enable researchers to obtain more reliable topologies in phylogenetic reconstructions and provide more opportunities to discover informative group-specific amino acids and/or nucleotides. Benefiting from these advances, we are now able to reframe the methodology of description in taxonomy. The hierarchical molecular apomorphy-based classification system proposed in this study can be very helpful in leading to precise descriptions of clades from the most microscopic but essential aspect of evolution and may even develop as an alternative, efficient approach for organism identification. Furthermore, the hierarchical apomorphy-based classification system provides a practical way of standardising the phylogenetic descriptions and nomenclature of clades, thus offering a potential methodological implementation of PhyloCode and facilitating its development. In this sense, the hierarchical apomorphy-based classification system can serve as a primer of integrative taxonomy^46^,^47^,^48^ linking phylogenomics, molecular classification, and phylogenetic nomenclature.

## Materials and Methods

### Apomorphy mining and sub-dataset generation

The transcriptome and/or genome data from a recent published phylogenomic study of Hexapoda^14^ and from a comparative genomic study of Anophelinae^15^ were chosen to generate two source datasets for this study. At the nucleotide level, due to evolutionary saturation and convergence, it is more common for two distantly related groups of organisms to share the same base status in a site, *i.e.*, molecular homoplasy. Therefore, contrary to previous strategies of molecular classification, we instead used the diversity of amino acids as clade description in this study. Considering the performance (signal versus noise) in the phylogenetic reconstruction, we chose the amino acid sequence matrix Supermatrix C_AA (provided by Misof *et al*.^14^) with a protein domain-based partitioning scheme as the source dataset for apomorphy search and named it Dataset 1. For the apomorphy mining of the genomes in the comparative genomic study by Neafsey *et al*.^15^, the matrix was constructed as follows. Seventeen genomes belonging to seven lineages in Anophelinae (Table S3) were downloaded from GenBank. Gene orthology was predicted using HaMStR v 13.2^49^ with the same core-orthologs as those used in the phylogenomic study of Misof *et al*.^14^. Sequences were then initially aligned using MAFFT^50^ and then refined using MUSCLE^51^. After the exclusion of random similar sites using ALICUT^52^ based on the score given by Aliscore^53^, sequences were concatenated as Dataset 2 using an in-house script.

Thereafter, we applied a parsimony method to explore potential apomorphies in the datasets. First, the sequence matrices and the corresponding treefiles were imported into PAUP* 4.0b10^54^. For Dataset 1, the topology of the treefile was fixed to the final result in the original study^14^. For Dataset 2, the topology of the treefile was the same as the one provided by the Broad Institute (https://olive.broadinstitute.org/projects/anopheles). All of the taxa in Hexapoda and Anophelinae were defined as ingroups. State optimisation of parsimony was set to DELTRAN. After the log-file option was activated, sequence data were then used to obtain a labelled tree with a complete list of apomorphies (Describetrees/root = outgroup plot = phylogram labelnode = yes apolist = yes). The results revealed all of the possible apomorphies of the corresponding dataset. Sites with ambiguous changes were abandoned. For species in distantly related groups, it is still possible that the evolution of amino acids also show convergence to some degree. To avoid this scenario, the CI of each apomorphy was then used as a filter criterion. Generally, the changes are very likely to be homoplasious if the value of CI is lower than 0.3. Herein, considering the randomness of the missing data in the majority of existing transcriptomic data, three more stringent filter criteria (CI varied from 1.0 to 0.8) were applied to ensure the credibility of the apomorphy call. A series of in-house shell scripts were used to filter the apomorphies listed in the log-file given by PAUP and to generate various sub-dataset files for the subsequent analyses, in which apomorphies filtered under different criteria were assembled.

### Phylogenetic analyses of the subsets

To compare the phylogenetic signal versus noise of apomorphies filtered by different criteria, three amino acid sequence sub-datasets for each of the two original datasets were generated, which were named sub-datasets 1A, 1B, 1C, and 2A, 2B, 2C, with the filter criterion of CI equal to or higher than 1.0, 0.9, and 0.8, respectively. For each sub-dataset, we performed phylogenetic analysis with maximum likelihood (ML) methods using the Pthreads-parallelised version of RAxML, v.8.0.12^55^. The provisional partition schemes were made with data blocks based on orthologous genes boundaries, and then PartitionFinder v. 1.1^56^ was used to infer further model estimation and partition schemes. As for sub-datasets trimmed from Dataset 1 and the corresponding reduced partition schemes, we used the same substitution model as the original one for consistency. The randomised stepwise addition parsimony trees were used as the starting tree. For each dataset, 100 searches were performed for the ML tree, with the robustness of branches tested by 100 bootstrap replicates. Rapid bootstrap analysis and the search for the best scoring ML tree were performed in a one program run (-f a).

### Design of a classification system of Hexapoda and Anophelinae using molecular apomorphies

According to its performance in the phylogenetic analyses, sub-dataset 1B in the case of Hexapoda and sub-dataset 2C in the case of Anophelinae were chosen for designing the clade codes. The principle described here consists of assigning each internal node in the phylogenetic tree a unique “code”, which represents the molecular apomorphy shared by that clade. Two classification systems corresponding to the category levels between superclass and order and between subfamily and species complex were constructed. Apomorphies of clades with extremely low coverage of taxon sampling were abandoned. The arrangements of codes were designed to be as hierarchical as possible, and they show a similar order within the tree obtained by phylogenetic inference. The following criteria were adopted to select a series of the most proper apomorphies from sub-datasets 1B and 2C.

1. Apomorphies for one clade should have no overlap with its sister-group, *i.e.*, if an apomorphic state of one clade is also found in its sister-group, such apomorphy should not be used because it is difficult to ascertain whether the possible apomorphy is a bona fide one or some type of plesiomorphy (Fig. 1a).
2. To ensure reliability, missing taxa that are supposed to share apomorphies should be limited. In addition, if the number of missing data is relatively large in the clade that is supposed to share the apomorphies, the reliability of the apomorphies becomes questionable. Apomorphies should never be used, no matter how “good”, if the states of the sister group or the basal clade of the analytic group are missing (ambiguous) (Fig. 1b).
3. Apomorphies that are unique, *i.e.*, autapomorphic, in a clade should be given the higher priority (Fig. 1a).
4. Apomorphies that are unique in the target clade are preferred. Apomorphies with binary or multinary states can also be candidates, but they should be less desirable unless they are shared by sub-clades and show a consistent evolutionary trend within the tree (Fig. 1c). For those preferred apomorphies, preference increases with the strength of the attributes that are close to autapomorphies (Fig. 1d).
5. For the purpose of simplification, it is more acceptable that fewer gene markers are involved. Therefore, the preference for a gene increases as the range of the taxa possessing a corresponding amino acid apomorphy broadens.

Following these criteria, we made an apomorphy-screening pipeline (Fig. S5) and selected one apomorphy for each node for the convenience of illustration. However, for groups that contain only one taxon in our sample (*e.g.*, Protura, Zoraptera and Mantophasmatodea), the apomorphies of these nodes were discarded because such characters are not shared by members within each taxon. The description of these apomorphic characters involves the state changes of each amino acid, the names of genes that possess those amino acid sites, the positions of those sites compared to their homologous genes in Drosophila, and the available structure information of the corresponding proteins, which were inferred with SWISS-Model online workplace^57^,^58^ and filtered by similarity (higher than 40%).

### Identification of unknown transcriptome as a proof-of-concept test

Considering the incompleteness of transcriptome data due to missing genes and missing sites in sequenced genes, it could be argued that transcriptomic data can be adequate for query identification. Therefore, we carried out a group of tests to see the identification ability of an unknown transcriptome based on diagnostic apomorphies. In view of the taxon coverage of transcriptome data, the sequence assemblies of 15 *de novo* sequenced transcriptomes in this study and 36 transcriptomes obtained through GenBank (and not used in the works of Misof *et al*.^14^), which together represent 18 orders of insects (Table S2), were used for querying the designed classification system of Hexapoda. The test results of Polyneoptera were additionally summarised considering both the taxon coverage of the dataset and the historical controversy.

The workflow of identification is illustrated in Fig. 5d. Contrary to the leaves-only processing of DNA through barcoding (Fig. 5c), the identification can be interpreted as a query along the lineage from the root to the leaf. The first step of identification is predicting the orthology of the queried transcriptome or genome using the core-orthologs of database, followed by alignment between the predicted orthologs and the core-orthologs. Subsequent identifications then follow a series of progressive steps corresponding to the fixed sequential steps as those in an identification key. If the matching judgment of a certain internal node based on the state of apomorphic amino acid is fulfilled, then the identification will progress and continue repeatedly until a terminal node is reached or no matches are found, at which point the final generated query description is obtained by assembling the matched codes in a hierarchical order. If the matching judgment encounters some missing state and the corresponding step is ambiguous, then the subsequent steps will progressively check the state of each child node until a clear judgment can be given (Fig. S6).

## Additional Information

**How to cite this article**: Wu, H.-Y. *et al*. Molecular classification based on apomorphic amino acids (Arthropoda, Hexapoda): Integrative taxonomy in the era of phylogenomics. *Sci. Rep.* **6**, 28308; doi: 10.1038/srep28308 (2016).

## Supplementary Material

Supplementary Information

## Figures and Tables

**Table 1:** Results of query test of the 51 unknown transcriptomes.

**Species**	**Result**	**Generated Barcode in Test**	**Designed Barcode (Database)**	**Category**
*Pantala flavescens fabricius*	Pterygota	T_00_I_02_X_03_E_06_R_08_X_09_X_0B_	T_00_I_02_G_03_E_06_R_08_S_09_C_0B_	False-negative
*Ischnura elegans*	Odonata	X_00_I_02_X_03_E_06_R_08_X_09_C_0B_	T_00_I_02_G_03_E_06_R_08_S_09_C_0B_	Positive
*Isonychia kiangsinensis*	Ephemeroptera	T_00_I_02_X_03_X_06_R_08_X_09_Q_0A_	T_00_I_02_G_03_E_06_R_08_S_09_Q_0A_	Positive
*Ephemera* sp.	Ephemeroptera	T_00_I_02_X_03_E_06_R_08_S_09_Q_0A_	T_00_I_02_G_03_E_06_R_08_S_09_Q_0A_	Positive
*Eparchus insignis*	Dermaptera	X_00_I_02_?_03_E_06_R_08_X_0C_Q_0D_H_0E_F_0F_	T_00_I_02_G_03_E_06_R_08_S_0C_Q_0D_H_0E_F_0F_	Positive
*Flavoperla* sp.	Plecoptera	X_00_I_02_X_03_?_06_R_08_X_0C_Q_0D_S_0G_	T_00_I_02_G_03_E_06_R_08_S_0C_Q_0D_S_0G_	Positive
*Chondracris rosea*	Orthoptera	T_00_I_02_X_03_E_06_R_08_S_0C_Q_0D_M_0H_	T_00_I_02_G_03_E_06_R_08_S_0C_Q_0D_M_0H_	Positive
*Gryllotalpa unispina*	Orthoptera	?_00_I_02_X_03_E_06_R_08_S_0C_Q_0D_M_0H_	T_00_I_02_G_03_E_06_R_08_S_0C_Q_0D_M_0H_	Positive
*Hymenopus coronatus*	Mantodea	X_00_I_02_X_03_E_06_R_08_S_0C_Q_0D_M_0I_H_0P_C_0R_	T_00_I_02_G_03_E_06_R_08_S_0C_Q_0D_M_0I_H_0P_C_0R_	Positive
*Phraortes* sp.	Phasmida	T_00_I_02_X_03_X_06_R_08_X_0C_Q_0D_M_0I_X_0J_L_0M_V_0O_	T_00_I_02_G_03_E_06_R_08_S_0C_Q_0D_M_0I_K_0J_L_0M_V_0O_	Positive
*Coptotermes formosanus*	Blattodea	T_00_I_02_X_03_E_06_R_08_S_0C_Q_0D_M_0I_H_0P_T_0S_	T_00_I_02_G_03_E_06_R_08_S_0C_Q_0D_M_0I_H_0P_T_0S_	Positive
*Eupolyphaga sinensis*	Blattodea	T_00_I_02_X_03_E_06_R_08_S_0C_Q_0D_M_0I_X_0P_T_0S_	T_00_I_02_G_03_E_06_R_08_S_0C_Q_0D_M_0I_H_0P_T_0S_	Positive
*Periplaneta Americana*	Blattodea	T_00_I_02_X_03_E_06_R_08_X_0C_Q_0D_M_0I_X_0P_T_0S_	T_00_I_02_G_03_E_06_R_08_S_0C_Q_0D_M_0I_H_0P_T_0S_	Positive
*Pedetontus* sp.	Archaeognatha	T_00_I_02_X_03_N_05_	T_00_I_02_G_03_N_05_	Positive
*Lepisma* sp.	Zygentoma	T_00_I_02_X_03_E_06_T_07_	T_00_I_02_G_03_E_06_T_07_	Positive
*Anoplophora glabripennis*	Coleopterodea	T_00_I_02_X_03_?_06_R_08_S_0C_X_0V_N_0X_N_0Z_R_10_H_16_X_18_	T_00_I_02_G_03_E_06_R_08_S_0C_I_0V_N_0X_N_0Z_R_10_H_16_K_18_	False negative
*Antheraea assama*	Lepidoptera	T_00_I_02_G_03_X_06_R_08_S_0C_X_0V_N_0X_N_0Z_N_19_L_1A_E_1C_	T_00_I_02_G_03_E_06_R_08_S_0C_I_0V_N_0X_N_0Z_N_19_L_1A_E_1C_	Positive
*Anthonomus grandis*	Coleoptera	?_00_I_02_X_03_E_06_R_08_X_0C_X_0V_N_0X_X_0Z_R_10_H_16_K_18_	T_00_I_02_G_03_E_06_R_08_S_0C_I_0V_N_0X_N_0Z_R_10_H_16_K_18_	Positive
*Bactrocera dorsalis*	Diptera	?_00_I_02_G_03_E_06_R_08_S_0C_I_0V_N_0X_N_0Z_N_19_A_1D_Y_1H_	T_00_I_02_G_03_E_06_R_08_S_0C_I_0V_N_0X_N_0Z_N_19_A_1D_Y_1H_	Positive
*Belgica antarctica*	Diptera	T_00_I_02_?_03_E_06_R_08_S_0C_I_0V_?_0X_N_0Z_N_19_A_1D_Y_1H_	T_00_I_02_G_03_E_06_R_08_S_0C_I_0V_N_0X_N_0Z_N_19_A_1D_Y_1H_	Positive
*Brassicogethes aeneus*	Coleoptera	T_00_I_02_G_03_E_06_R_08_S_0C_X_0V_N_0X_N_0Z_R_10_X_16_K_18_	T_00_I_02_G_03_E_06_R_08_S_0C_I_0V_N_0X_N_0Z_R_10_H_16_K_18_	Positive
*Chrysopa pallens*	Neuroptera	T_00_I_02_G_03_E_06_R_08_S_0C_X_0V_N_0X_N_0Z_R_10_M_11_X_13_C_15_	T_00_I_02_G_03_E_06_R_08_S_0C_I_0V_N_0X_N_0Z_R_10_M_11_M_13_C_15_	Positive
*Colaphellus bowringi*	Coleoptera	T_00_I_02_G_03_E_06_R_08_S_0C_I_0V_N_0X_N_0Z_R_10_H_16_K_18_	T_00_I_02_G_03_E_06_R_08_S_0C_I_0V_N_0X_N_0Z_R_10_H_16_K_18_	Positive
*Corydalinae* sp.	Megaloptera	T_00_I_02_X_03_?_06_R_08_X_0C_X_0V_X_0X_N_0Z_R_10_X_11_X_13_R_14_	T_00_I_02_G_03_E_06_R_08_S_0C_I_0V_N_0X_N_0Z_R_10_M_11_M_13_R_14_	Positive
*Crioscolia alcione*	Hymenoptera	T_00_I_02_G_03_E_06_R_08_S_0C_X_0V_N_0X_L_0Y_	T_00_I_02_G_03_E_06_R_08_S_0C_I_0V_N_0X_L_0Y_	Positive
*Culicoides* sp.	Diptera	T_00_I_02_G_03_E_06_R_08_?_0C_I_0V_N_0X_N_0Z_N_19_A_1D_Y_1H_	T_00_I_02_G_03_E_06_R_08_S_0C_I_0V_N_0X_N_0Z_N_19_A_1D_Y_1H_	Positive
*Dastarcus helophoroides*	Coleoptera	T_00_I_02_X_03_E_06_R_08_S_0C_X_0V_N_0X_N_0Z_R_10_H_16_K_18_	T_00_I_02_G_03_E_06_R_08_S_0C_I_0V_N_0X_N_0Z_R_10_H_16_K_18_	Positive
*Delia antiqua*	Diptera	T_00_I_02_?_03_E_06_R_08_S_0C_I_0V_N_0X_N_0Z_N_19_A_1D_Y_1H_	T_00_I_02_G_03_E_06_R_08_S_0C_I_0V_N_0X_N_0Z_N_19_A_1D_Y_1H_	Positive
*Fopius arisanus*	Hymenoptera	T_00_X_02_G_03_E_06_?_08_X_0C_I_0V_N_0X_L_0Y_	T_00_I_02_G_03_E_06_R_08_S_0C_I_0V_N_0X_L_0Y_	Positive
*Hypothenemus hampei*	Coleoptera	T_00_I_02_?_03_E_06_?_08_?_0C_?_0V_N_0X_?_0Z_R_10_H_16_K_18_	T_00_I_02_G_03_E_06_R_08_S_0C_I_0V_N_0X_N_0Z_R_10_H_16_K_18_	Positive
*Ips typographus*	Coleoptera	T_00_X_02_?_03_?_06_R_08_?_0C_X_0V_?_0X_X_0Z_R_10_X_16_K_18_	T_00_I_02_G_03_E_06_R_08_S_0C_I_0V_N_0X_N_0Z_R_10_H_16_K_18_	Positive
*Lymantria dispar*	Lepidoptera	T_00_I_02_?_03_X_06_R_08_X_0C_X_0V_N_0X_X_0Z_N_19_L_1A_E_1C_	T_00_I_02_G_03_E_06_R_08_S_0C_I_0V_N_0X_N_0Z_N_19_L_1A_E_1C_	Positive
*Musca domestica*	Diptera	T_00_I_02_G_03_E_06_R_08_S_0C_X_0V_X_0X_N_0Z_N_19_A_1D_Y_1H_	T_00_I_02_G_03_E_06_R_08_S_0C_I_0V_N_0X_N_0Z_N_19_A_1D_Y_1H_	Positive
*Nevrorthus apatelios*	Neuroptera	?_00_?_02_?_03_X_06_?_08_?_0C_X_0V_?_0X_?_0Z_R_10_?_11_X_13_C_15_	T_00_I_02_G_03_E_06_R_08_S_0C_I_0V_N_0X_N_0Z_R_10_M_11_M_13_C_15_	Positive
*Nicrophorus vespilloides*	Coleoptera	T_00_I_02_G_03_E_06_R_08_S_0C_I_0V_N_0X_N_0Z_R_10_H_16_K_18_	T_00_I_02_G_03_E_06_R_08_S_0C_I_0V_N_0X_N_0Z_R_10_H_16_K_18_	Positive *
*Oropsylla silantiewi*	Siphonaptera	T_00_I_02_G_03_E_06_R_08_S_0C_I_0V_N_0X_N_0Z_N_19_A_1D_G_1E_	T_00_I_02_G_03_E_06_R_08_S_0C_I_0V_N_0X_N_0Z_N_19_A_1D_G_1E_	Positive *
*Osmia cornuta*	Hymenoptera	T_00_I_02_G_03_E_06_R_08_X_0C_X_0V_N_0X_L_0Y_	T_00_I_02_G_03_E_06_R_08_S_0C_I_0V_N_0X_L_0Y_	Positive
*Polistes metricus*	Hymenoptera	T_00_I_02_G_03_E_06_R_08_S_0C_I_0V_X_0X_L_0Y_	T_00_I_02_G_03_E_06_R_08_S_0C_I_0V_N_0X_L_0Y_	Positive
*Raphidia ariadne*	Raphidioptera	T_00_X_02_G_03_?_06_R_08_X_0C_X_0V_?_0X_X_0Z_?_10_X_11_N_12_	T_00_I_02_G_03_E_06_R_08_S_0C_I_0V_N_0X_N_0Z_R_10_M_11_N_12_	Positive
*Rhodinia newara*	Lepidoptera	T_00_I_02_G_03_E_06_R_08_S_0C_X_0V_N_0X_N_0Z_N_19_L_1A_E_1C_	T_00_I_02_G_03_E_06_R_08_S_0C_I_0V_N_0X_N_0Z_N_19_L_1A_E_1C_	Positive
*Samia ricini*	Lepidoptera	T_00_I_02_G_03_X_06_R_08_S_0C_X_0V_N_0X_X_0Z_N_19_L_1A_E_1C_	T_00_I_02_G_03_E_06_R_08_S_0C_I_0V_N_0X_N_0Z_N_19_L_1A_E_1C_	Positive
*Sitodiplosis mosellana*	Diptera	?_00_I_02_G_03_?_06_R_08_S_0C_I_0V_N_0X_N_0Z_N_19_?_1D_Y_1H_	T_00_I_02_G_03_E_06_R_08_S_0C_I_0V_N_0X_N_0Z_N_19_A_1D_Y_1H_	Positive
*Stomoxys calcitrans*	Diptera	T_00_I_02_G_03_E_06_R_08_S_0C_I_0V_N_0X_N_0Z_N_19_A_1D_Y_1H_	T_00_I_02_G_03_E_06_R_08_S_0C_I_0V_N_0X_N_0Z_N_19_A_1D_Y_1H_	Positive *
*Telchin licus*	Lepidoptera	?_00_?_02_G_03_X_06_R_08_X_0C_X_0V_?_0X_?_0Z_N_19_?_1A_E_1C_	T_00_I_02_G_03_E_06_R_08_S_0C_I_0V_N_0X_N_0Z_N_19_L_1A_E_1C_	Positive
*Telenomus podisi*	Hymenoptera	T_00_X_02_?_03_E_06_R_08_S_0C_X_0V_N_0X_L_0Y_	T_00_I_02_G_03_E_06_R_08_S_0C_I_0V_N_0X_L_0Y_	Positive
*Teleopsis whitei*	Diptera	T_00_I_02_G_03_E_06_R_08_S_0C_I_0V_N_0X_N_0Z_N_19_A_1D_Y_1H_	T_00_I_02_G_03_E_06_R_08_S_0C_I_0V_N_0X_N_0Z_N_19_A_1D_Y_1H_	Positive *
*Tetramorium bicarinatum*	Hymenoptera	T_00_I_02_?_03_E_06_R_08_S_0C_X_0V_N_0X_L_0Y_	T_00_I_02_G_03_E_06_R_08_S_0C_I_0V_N_0X_L_0Y_	Positive
*Thaumetopoea pityocampa*	Lepidoptera	?_00_?_02_?_03_?_06_R_08_?_0C_?_0V_X_0X_?_0Z_N_19_X_A_E_1C_	T_00_I_02_G_03_E_06_R_08_S_0C_I_0V_N_0X_N_0Z_N_19_L_1A_E_1C_	Positive
*Themira biloba*	Diptera	T_00_I_02_G_03_E_06_R_08_S_0C_I_0V_N_0X_N_0Z_N_19_A_1D_Y_1H_	T_00_I_02_G_03_E_06_R_08_S_0C_I_0V_N_0X_N_0Z_N_19_A_1D_Y_1H_	Positive *
*Xyela alpigena*	Holometabola	?_00_?_02_?_03_X_06_X_08_?_0C_X_0V_N_0X_?_0Y_	T_00_I_02_G_03_E_06_R_08_S_0C_I_0V_N_0X_L_0Y_	False-negative
*Yponomeuta evonymellus*	Lepidoptera	X_00_I_02_X_03_X_06_R_08_S_0C_X_0V_N_0X_X_0Z_N_19_L_1A_E_1C_	T_00_I_02_G_03_E_06_R_08_S_0C_I_0V_N_0X_N_0Z_N_19_L_1A_E_1C_	Positive

“X” represents missing amino acid residues, while “?” represents missing gene. “*” represents complete-matching result.
