# Supplementary material for: Molecular classification based on apomorphic amino acids (Arthropoda, Hexapoda): Integrative taxonomy in the era of phylogenomics
Source: Sci Rep. 2016 Jun 17;6:28308. doi: 10.1038/srep28308 (PMC4911608; doi:10.1038/srep28308)
Supplement: Supplementary Information [file srep28308-s1.pdf]

Supplementary Information

Molecular classification based on apomorphic amino acids (Arthropoda, Hexapoda): Integrative taxonomy in the era of phylogenomics

Hao-Yang Wu[1†], Yan-Hui Wang[1,2†], Qiang Xie[1†*], Yun-Ling Ke[3], Wen-Jun Bu[1].

[1]*Institute of Entomology, College of Life Sciences, Nankai University, Tianjin 300071, China*.

[2]*College of Computer and Control Engineering, Nankai University, 38 Tongyan Road, Haihe Education Park, Jinnan District, Tianjin 300350, China.*

[3]*Guangdong Entomological Institute, Guangzhou 510260, China*.

† These authors contributed equally to this work.

*Corresponding Author: qiangxie@nankai.edu.cn (Q.X.)

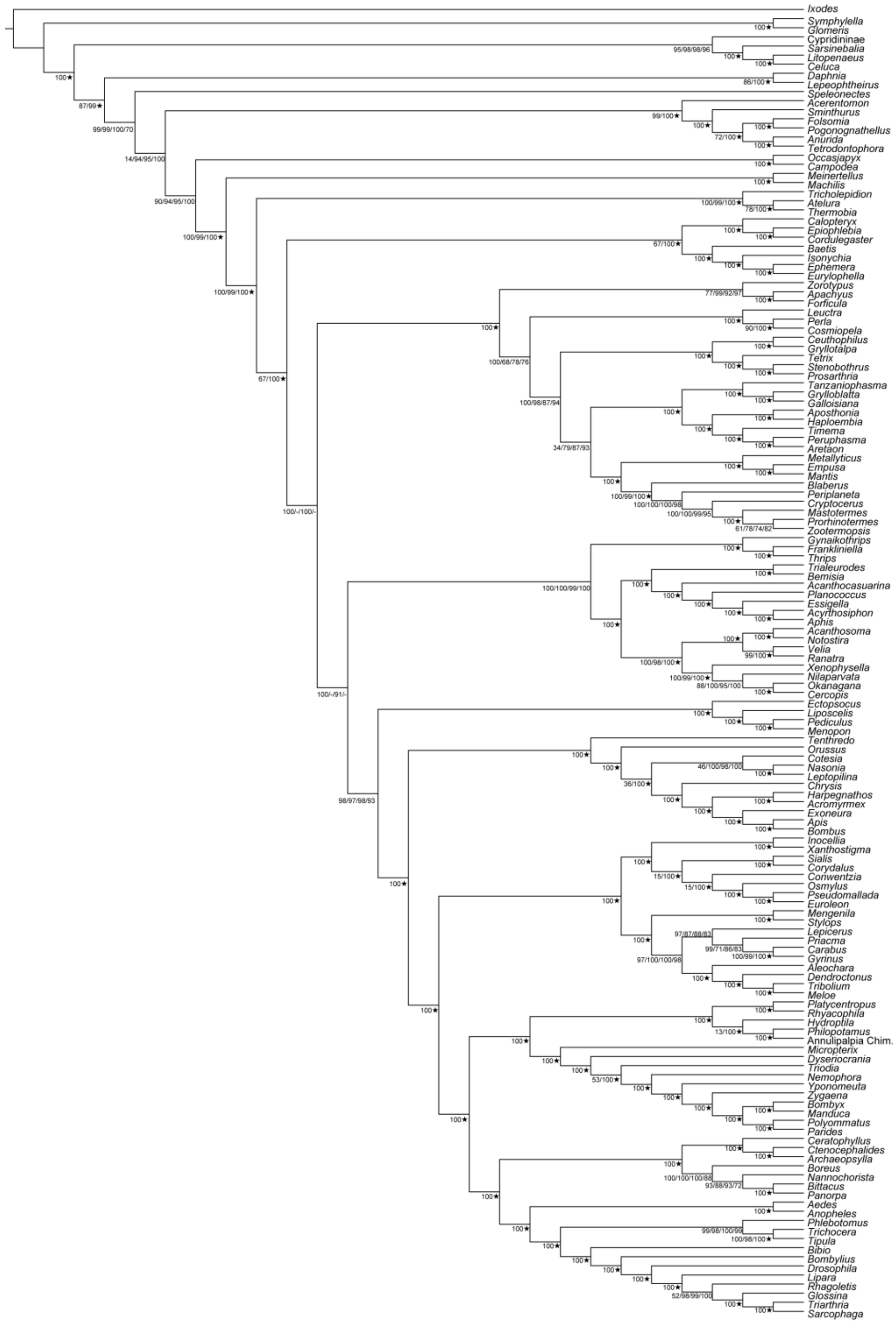

Figure S1. Summarized cladogram based on sub-dataset 1A, 1B, 1C and the original supermatrices. Branch support values are shown, following the order of original/CI 1.0/ CI 0.9/CI 0.8. The black star indicates that the following bootstrap values are the same as the former one. The dash shown in the bootstrap values indicates that the monophyly of that clade is not supported in the respective dataset.

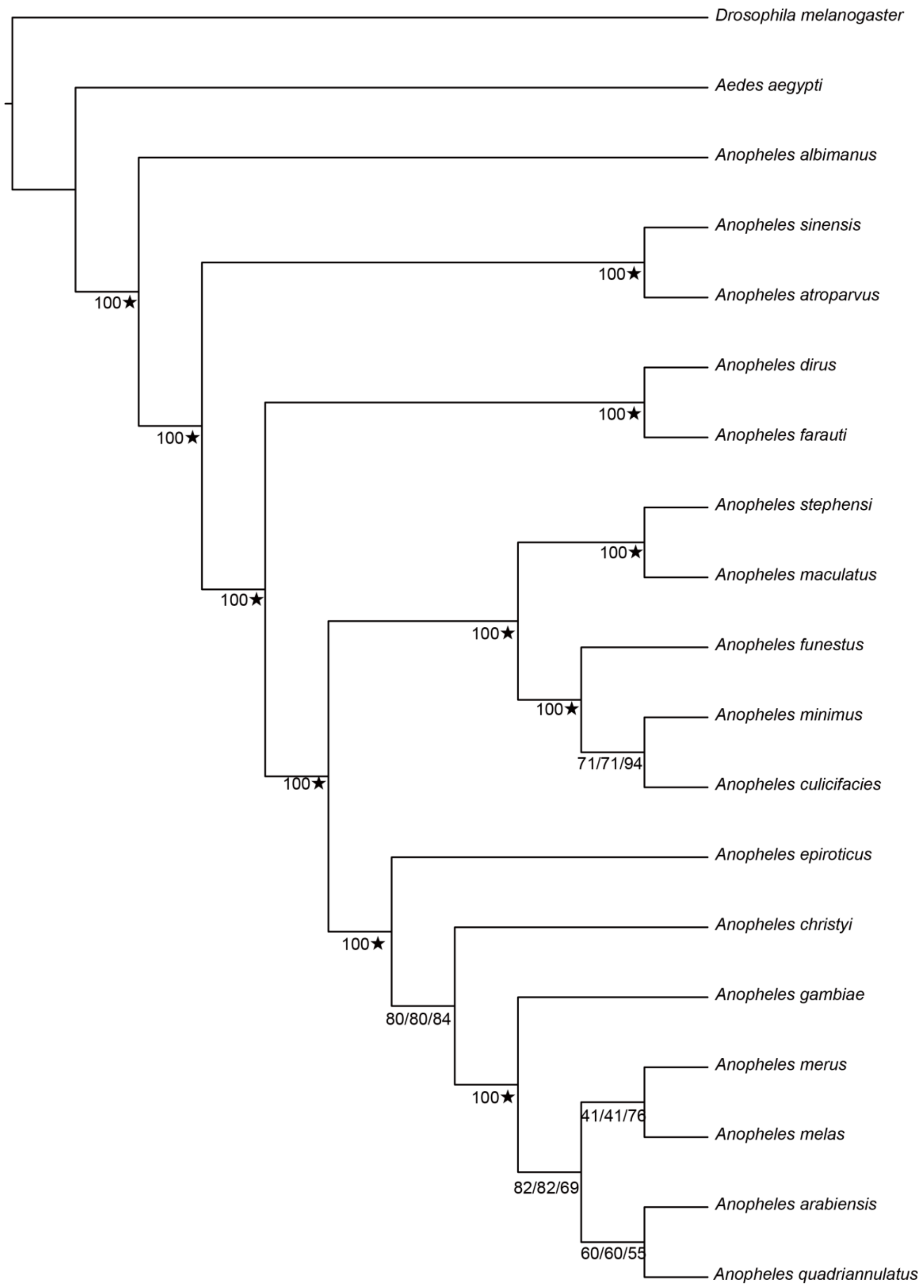

Figure S2. **Summary cladogram of sub-dataset 2A, 2B, and 2C.** Branch support values are shown, following the order of CI 1.0/ CI 0.9/CI 0.8. The black star indicates that the following bootstrap values are the same as the former one.

Column	Gene	Site	Structure
00	Snf5-related 1	258	Not availiable
01	Inosine triphosphate pyrophosphatase	111	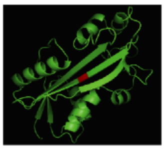
02	Clathrin heavy chain	829	Not availiable
03	Nuclear hormone receptor FTZ-F1	961	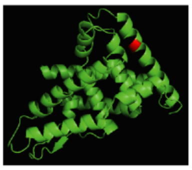
04	40S ribosomal protein S20	64	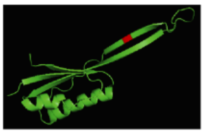
05	40S ribosomal protein S3A	155	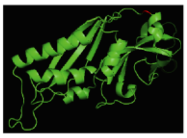
06	Integrin linked kinase	368	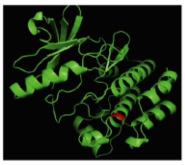
07	ATP-dependent RNA helicase abstrakt	312	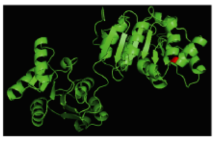
08	Mediator of RNA polymerase II transcription subunit 7	76	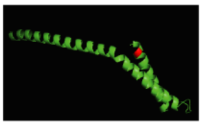
09	Mi-2	1454	Not availiable
0A	Eukaryotic translation initiation factor 3 subunit C	645	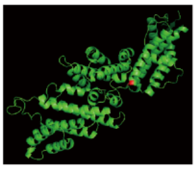
0B	Eukaryotic translation initiation factor 3 subunit C	478	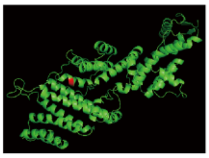

0C	Vacuolar protein sorting 35	298	Not avaliable
0D	Casein kinase II subunit beta	165	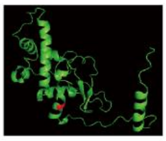
0E	RE55033p	178	Not avaliable
0F	Kelch-like protein diablo	437	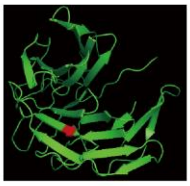
0G	Amphiphysin	178	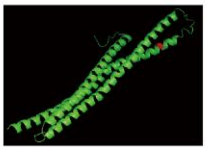
0H	ATP-dependent RNA helicase Ddx1	26	Not avaliable
0I	Probable prefoldin subunit 4	120	Not avaliable
0J	Trailer hitch	33	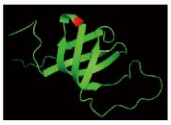
0K	Integrin linked kinase	395	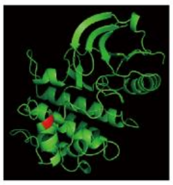
0L	Eukaryotic translation initiation factor 3 subunit L	440	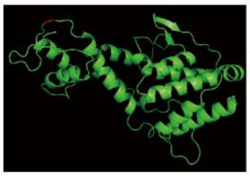
0M	NADH:ubiquinone reductase 42kD subunit	147	Not avaliable
0N	RuvB-like helicase 2	126	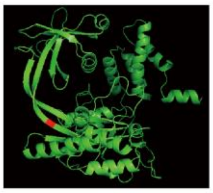
0O	Tyrosyl-tRNA synthetase	435	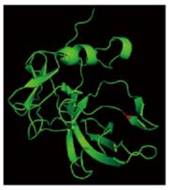
0P	Transcription initiation factor IIA subunit 1	180	Not avaliable

0Q	RuvB-like helicase 2	207	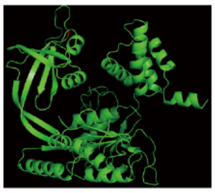
0R	Probable cysteine desulfurase	206	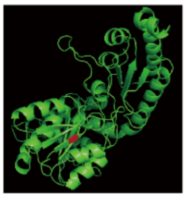
0S	Hormone receptor 4	1288	Not avaliable
0T	Protein suppressor of forked	455	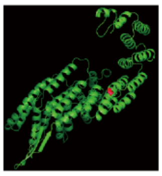
0U	Ras-related GTP binding C	62	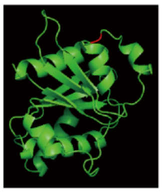
0V	Protein suppressor of forked	96	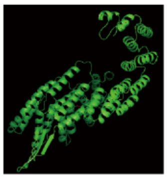
0W	Mi-2	947	Not avaliable
0X	Ef1alpha-like factor	606	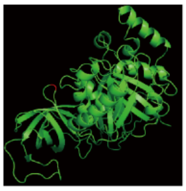
0Y	NEDD8-activating enzyme E1 regulatory subunit	490	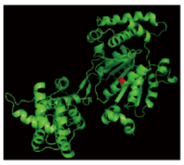
0Z	Zn72D	559	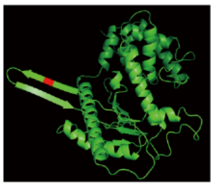
10	CG34133	998	Not avaliable
11	Xeroderma pigmentosum D	555	Not avaliable

12	RPL23A	210	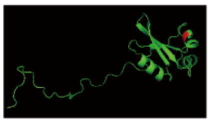
13	RAC serine/threonine-protein kinase	303	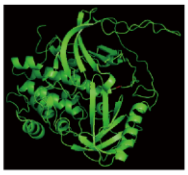
14	CG5451	339	Not avaliable
15	Ragulator complex protein LAMTOR2	36	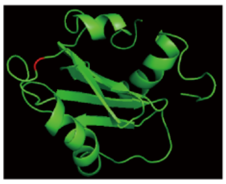
16	Asparaginyl-tRNA synthetase	554	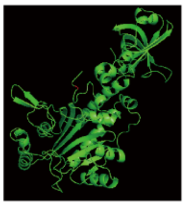
17	40S ribosomal protein S20	38	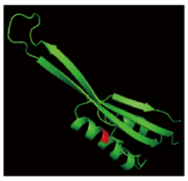
18	RNA polymerase II p33 subunit	51	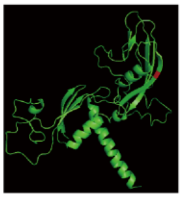
19	Eukaryotic translation initiation factor 3 subunit M	114	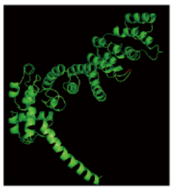
1A	Coatomer subunit beta'	408	Not avaliable
1B	Eukaryotic translation initiation factor 3 subunit M	328	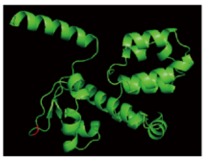
1C	Mitochondrial ribosomal protein S14	79	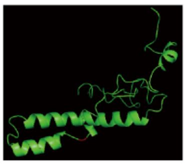

1D	Xeroderma pigmentosum D, isoform A	598	Not avaliable
1E	Probable elongator complex protein 3	458	Not avaliable
1F	Spliceosomal protein on the X	214	Not avaliable
1G	Leucine-rich repeat protein soc-2 homolog	168	Not avaliable
1H	Ubiquitin carboxyl-terminal hydrolase	692	Not avaliable
S01	Alpha-coatomer protein	950	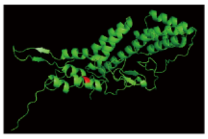
S02	Alpha-coatomer protein	954	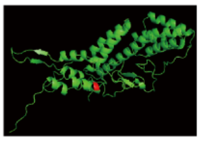
S03	CG11771	207	Not avaliable
S04	Alpha-coatomer protein	1128	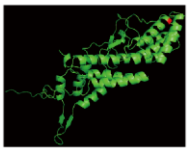
S05	ATP-dependent RNA helicase abstrakt	239	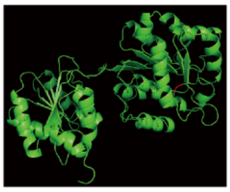
S06	Putative U5 small nuclear ribonucleoprotein 200 kDa helicase	1871	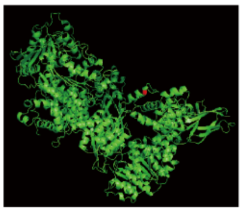
S07	Alpha-coatomer protein	501	Not avaliable
S08	Protein shuttle craft	744	Not avaliable
S09	Alpha-coatomer protein	987	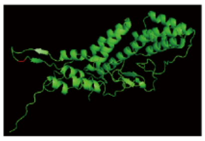
S0A	Putative U5 small nuclear ribonucleoprotein 200 kDa helicase	1626	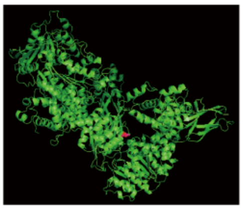

Figure S3. The annotations of IDs used in the classification system. The apomorphic sites are colored red in the three-dimensional structures.

(a)

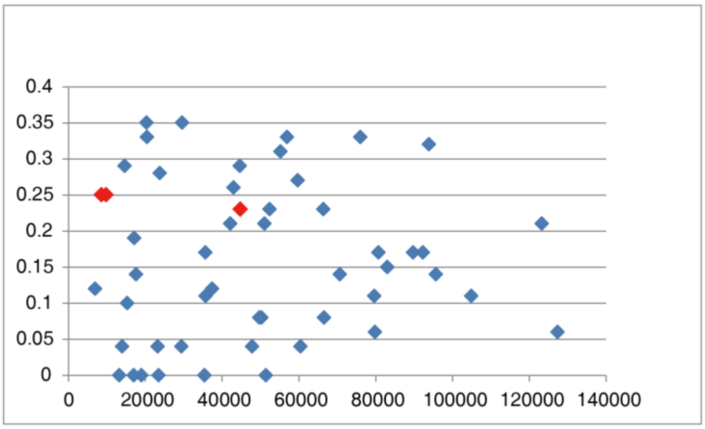

(b)

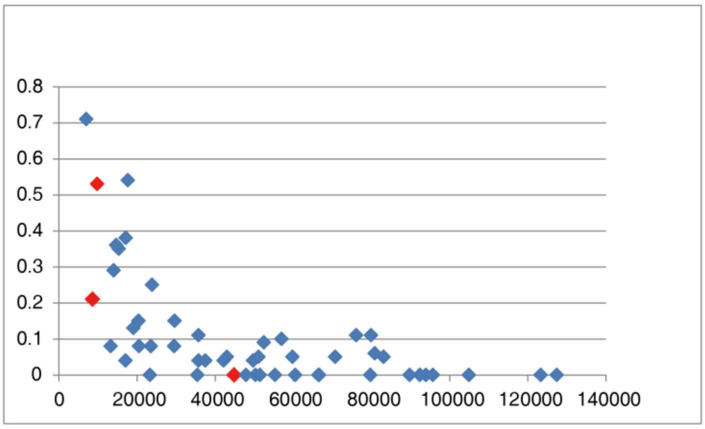

(c)

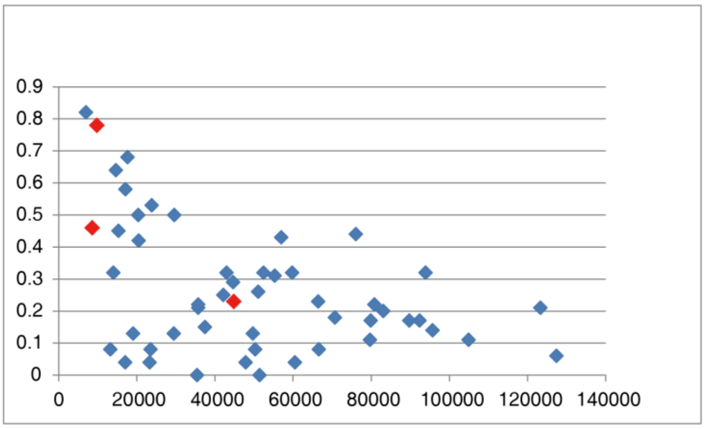

Figure S4. Scatter diagram about the proportion of missing states to the size of tested transcriptome. The dots in red represent the transcriptomes with a false-negative results, while the dots in blue represent the transcriptomes with positive results. The horizontal axis represents the size of transcriptomes, while the longitudinal axis represents the proportion of: (a) missing amino acid residues. (b) missing genes. (c) missing states.

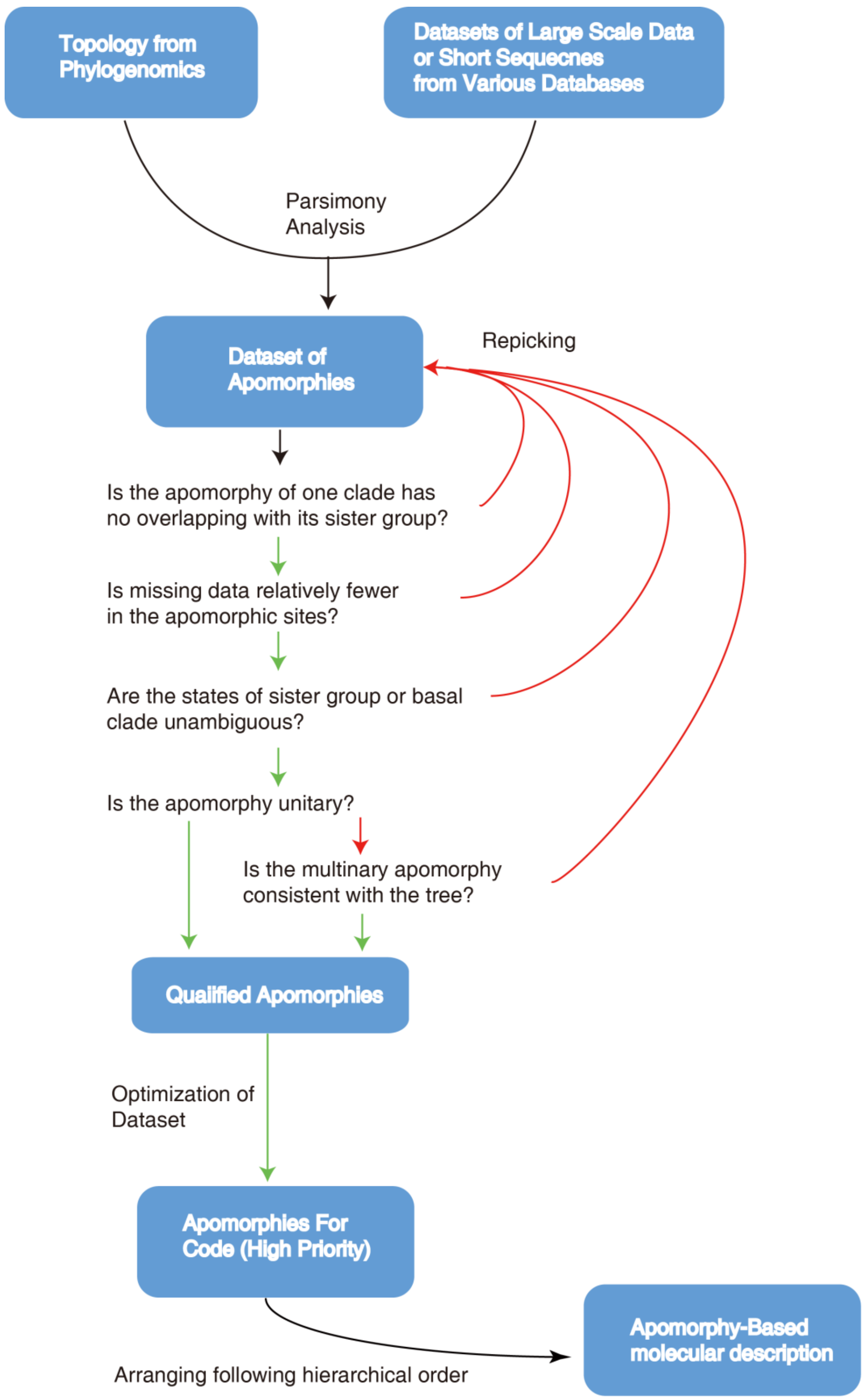

Figure S5. General pipeline for apomorphy screening. The green line indicates that the criterion is fulfilled, while the red line indicates the criterion is not fulfilled.

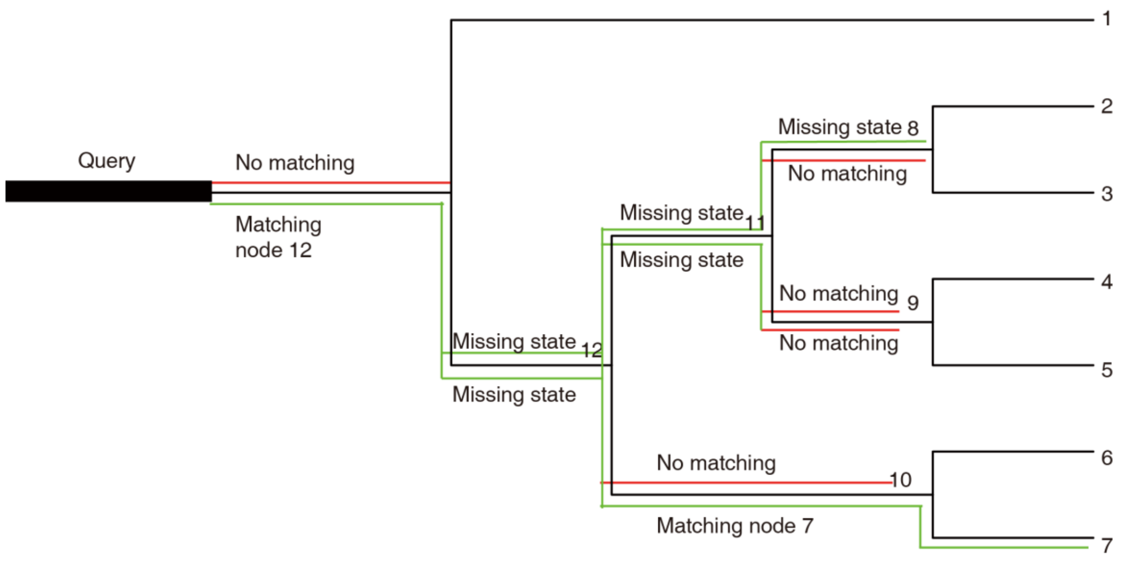

Figure S6. General pipeline for identification in this system. The green line indicates that the identification can make progress along this branch, while the red line indicates identification cannot make progress along this branch.

Table S1. Full test results of the 51 queried transcriptomes.

Organism	00	01	02	03	04	05	06	07	08	09	0A	0B	0C	0D	0E	0F	0G
Pantala flavescens fabricius	I	D	I	M	D	D	I	D	I	M	D	M	D				
Ischnura elegans	M	D	I	M	D	D	I	D	M	M	D	I	D	D			
Isonychia kiangsinensis	I	D	I	M	D	D	M	D	I	M	I	D	D				
Ephemera sp.	I	D	I	M	D	D	I	D	I	I	I	D	D				
Eparchus insignis	M	M	I	M	D	D	I	D	I	D			M	I	I	I	D
Flavoperla sp.	M	D	I	M	D	D	M	D	I	D			M	I	M		I
Chondracris rosea	I	D	I	M	D	D	I	D	I	D			I	I	D		D
Gryllotalpa unispina	M	D	I	M	D	D	I	D	I	D			I	I	D		D
Hymenopus coronatus	M	D	I	M	D	D	M	D	I	D			M	I	D		D
Phraortes sp.	I	D	I	M	D	D	I	D	I	D			M	I	D		D
Coptotermes formosanus	I	D	I	M	D	D	I	D	I	D			I	I	M		D
Eupolyphaga sinensis	I	M	I	M	D	D	I	M	I	M			I	I	M		D
Periplaneta americana	I	D	I	M	D	D	I	M	I	D			M	I	M		D
Pedetontus sp.	I	D	I	M	D	I	D										
Lepisma sp.	I	D	I	M	D	D	I	I	D								
Anoplophora glabripennis	I	D	I	M	D	M	M	M	I	M			I	D			
Antheraea assama	I	D	I	I	D	D	M	D	I	D			I	D			
Anthonomus grandis	M	D	M	M	D	D	I	M	I	M	D	D	M	M			
Bactrocera dorsalis	M	D	I	I	D	D	I	D	I	D			I	D			
Belgica antarctica	I	D	I	M	D	D	I	D	I	D			I	D			
Brassicogethes aeneus	I	D	I	I	D	D	I	D	I	D			I	D			
Chrysopa pallens	I	D	I	I	D	D	I	D	I	D			I	D			
Colaphellus bowringi	I	D	I	I	D	D	I	D	I	D			I	D			
Corydalinae sp.	I	D	M	I	D	D	M	M	I	D			M	D			
Crioscolia alcione	I	D	I	I	D	M	I	D	I	D			I	D			
Culicoides sp.	I	M	I	I	M	D	I	D	I	D			M	D			
Dastarcus helophoroides	I	D	I	M	D	D	I	D	I	M			I	D			
Delia antiqua	I	D	I	M	D	D	I	D	I	D			I	D			
Fopius arisanus	I	D	M	I	M	M	I	D	M	M	M	M	M	D			
Hypothenemus hampei	I	D	I	M	M	D	I	D	M	M	M	M	M	M			
Ips typographus	I	D	M	M	M	D	M	M	I	D			M	M			
Lymantria dispar	I	M	I	M	D	D	M	D	I	D			M	D			
Musca domestica	I	D	I	I	D	D	I	D	I	D			I	D			
Nevrorthus apatelios	M	D	M	M	D	D	M	M	M	M	M	M	M	D			
Nicrophorus vespilloides	I	M	I	I	D	D	I	D	I	D			I	D			
Oropsylla silantiewi	I	D	I	I	D	D	I	D	I	D			I	M			
Osmia cornuta	I	D	I	I	M	D	I	D	I	M	D	D	M	D			
Polistes metricus	I	D	I	I	D	D	I	D	I	D			I	D			
Raphidia ariadne	I	D	M	I	D	M	M	D	I	M	D	D	M	D			
Rhodinia newara	I	D	I	I	D	D	I	D	I	D			I	D			
Samia ricini	I	M	I	I	D	M	M	D	I	D			I	D			
Sitodiplosis mosellana	M	D	I	I	D	D	M	M	I	D			I	D			
Stomoxys calcitrans	I	D	I	I	D	D	I	D	I	D			I	D			
Telchin licus	M	D	M	I	D	D	M	M	I	M	D	D	M	M			
Telenomus podisi	I	D	M	M	D	D	I	D	I	D			I	D			
Teleopsis whitei	I	D	I	I	D	M	I	D	I	D			I	D			
Tetramorium bicarinatum	I	D	M	I	M	M	I	D	I	D			I	D			
Thaumetopoea pityocampa	M	M	M	M	M	D	M	M	I	M	M	M	M	M			
Themira biloba	I	D	I	I	D	D	I	M	I	D			I	D			
Xyela alpigena	M	M	M	M	D	D	M	M	M	M	M	M	M	D			
Yponomeuta evonymellus	M	D	I	M	D	D	M	D	I	D			I	D			

Organism	0H	0I	0J	0K	0L	0M	0N	0O	0P	0Q	0R	0S	0T	0U	0V	0W	0X
Pantala flavescens fabricius																	
Ischnura elegans												D			M		
Isonychia kiangsinensis																	
Ephemera sp.																	
Eparchus insignis	M	D										D			M		
Flavoperla sp.	M	D										M			M		
Chondracris rosea	I	D										D			M		
Gryllotalpa unispina	I	D										M			M		
Hymenopus coronatus	D	M	M						I	I	D	D			M		
Phraortes sp.	D	I	M	D		I	D	I	D			D			M		
Coptotermes formosanus	D	I	D						I	D	I	M			M		
Eupolyphaga sinensis	D	I	D						M	D	I	D			M		
Periplaneta americana	D	I	D						M	D	I	M			M		
Pedetontus sp.																	
Lepisma sp.																	
Anoplophora glabripennis												M			M	M	I
Antheraea assama												M			M	D	I
Anthonomus grandis												M			M	M	I
Bactrocera dorsalis												M			I	D	I
Belgica antarctica												D			I	D	M
Brassicogethes aeneus												M			M	D	I
Chrysopa pallens												D			M	D	I
Colaphellus bowringi												D			I	D	I
Corydalinae sp.												M			M	M	M
Crioscolia alcione												M			M	D	I
Culicoides sp.												D			I	D	I
Dastarcus helophoroides												M			M	D	I
Delia antiqua												M			I	M	I
Fopius arisanus												D			I	M	I
Hypothenemus hampei												M			M	M	I
Ips typographus												M			M	M	M
Lymantria dispar												M			M	M	I
Musca domestica												D			M	M	M
Nevrorthus apatelios												M			M	M	M
Nicrophorus vespilloides												D			I	D	I
Oropsylla silantiewi												D			I	D	I
Osmia cornuta												D			M	D	I
Polistes metricus												D			I	D	M
Raphidia ariadne												M			M	M	M
Rhodinia newara												M			M	D	I
Samia ricini												D			M	D	I
Sitodiplosis mosellana												D			I	D	I
Stomoxys calcitrans												D			I	D	I
Telchin licus												M			M	M	M
Telenomus podisi												M			M	D	I
Teleopsis whitei												D			I	D	I
Tetramorium bicarinatum												D			M	D	I
Thaumetopoea pityocampa												M			M	M	M
Themira biloba												D			I	D	I
Xyela alpigena												M			M	M	I
Yponomeuta evonymellus												D			M	D	I

Organism	0Y	0Z	10	11	12	13	14	15	16	17	18	19	1A	1B	1C	1D	1E
Pantala flavescens fabricius																	
Ischnura elegans																	
Isonychia kiangsinensis																	
Ephemera sp.																	
Eparchus insignis																	
Flavoperla sp.																	
Chondracris rosea																	
Gryllotalpa unispina																	
Hymenopus coronatus																	
Phraortes sp.																	
Coptotermes formosanus																	
Eupolyphaga sinensis																	
Periplaneta americana																	
Pedetontus sp.																	
Lepisma sp.																	
Anoplophora glabripennis	M	I	I	M					I	D	M	D					
Antheraea assama	M	I	D									I	I	D	I	M	
Anthonomus grandis	D	M	I	M					I	D	I	M					
Bactrocera dorsalis	D	I	D									I	D			I	D
Belgica antarctica	D	I	D									I	D			I	D
Brassicogethes aeneus	D	I	I	D					M	D	I	M	D			D	
Chrysopa pallens	D	I	I	I	D	M	D	I	D			D					
Colaphellus bowringi	D	I	I	d					I	D	I	D					
Corydalinae sp.	M	I	I	M	D	M	I	D	D			M	M	D	D	D	
Crioscolia alcione	I	D															
Culicoides sp.	D	I	D									I	D			I	D
Dastarcus helophoroides	M	I	I	M					I	D	I	D					
Delia antiqua	D	I	D									I	D			I	D
Fopius arisanus	I	D															
Hypothenemus hampei	M	I	M	M					I	M	I	D					
Ips typographus	M	M	I	M	M	M	M	D	M	D	I	D					
Lymantria dispar	D	M	M									I	I	D	I	M	
Musca domestica	M	I	D									I	D			I	D
Nevrorthus apatelios	D	M	I	M	D	M	M	I	D			M					
Nicrophorus vespilloides	D	I	I	D					I	D	I	D					
Oropsylla silantiewi	D	I	D									I	D			I	I
Osmia cornuta	I	D															
Polistes metricus	I	D															
Raphidia ariadne	D	M	M	M	I	M			M	D	D	M	M	D	D	M	D
Rhodinia newara	D	I	D									I	I	D	I	M	
Samia ricini	M	M	D									I	I	D	I	M	
Sitodiplosis mosellana	D	I	D									I	M	D	M	M	M
Stomoxys calcitrans	D	I	D									I	D			I	D
Telchin licus	D	M	M	M	D	D			M	D	D	I	M	D	I	M	
Telenomus podisi	I	M	D														
Teleopsis whitei	D	I	D									I	D			I	D
Tetramorium bicarinatum	I	D															
Thaumetopoea pityocampa	M	M	M	M	M	M	M	D	M	M	D	I	M	M	I	M	
Themira biloba	D	I	D									I	D				D
Xyela alpigena	M	M	M	M	D	M	M	M	D			M	M	D	D	M	M
Yponomeuta evonymellus	D	M	D									I	I	D	I	D	

Organism	1F	1G	1H
Pantala flavescens fabricius			
Ischnura elegans			
Isonychia kiangsinensis			
Ephemera sp.			
Eparchus insignis			
Flavoperla sp.			
Chondracris rosea			
Gryllotalpa unispina			
Hymenopus coronatus			
Phraortes sp.			
Coptotermes formosanus			
Eupolyphaga sinensis			
Periplaneta americana			
Pedetontus sp.			
Lepisma sp.			
Anoplophora glabripennis			
Antheraea assama			
Anthonomus grandis			
Bactrocera dorsalis			I
Belgica antarctica			I
Brassicogethes aeneus			
Chrysopa pallens			
Colaphellus bowringi			
Corydalinae sp.			
Crioscolia alcione			
Culicoides sp.			I
Dastarcus helophoroides			
Delia antiqua			I
Fopius arisanus			
Hypothenemus hampei			
Ips typographus			
Lymantria dispar			
Musca domestica			I
Nevrorthus apatelios			
Nicrophorus vespilloides			
Oropsylla silantiewi	D	I	
Osmia cornuta			
Polistes metricus			
Raphidia ariadne			D
Rhodinia newara			
Samia ricini			
Sitodiplosis mosellana	M	M	I
Stomoxys calcitrans			I
Telchin licus			
Telenomus podisi			
Teleopsis whitei			I
Tetramorium bicarinatum			
Thaumetopoea pityocampa			
Themira biloba			I
Xyela alpigena	M	M	M
Yponomeuta evonymellus			

I=identical, D=different, M=missing residues, MG=missing genes

Table S2. Accession number of the 51 queried transcriptomes.

Organism	Accession
Pedetontus sp. *	SRP039567
Lepisma sp. *	SRP039462
Pantala flavescens *	SRP039465
Ischnura elegans *	SRP039470
Isonychia kiangsinensis *	SRP039476
Ephemera sp. *	SRP039566
Eparchus insignis *	SRP039568
Flavoperla sp. *	SRP039570
Chondracris rosea *	SRP039466
Gryllotalpa unispina *	SRP039569
Hymenopus coronatus *	SRP039574
Phraortes sp. *	SRP040043
Coptotermes formosanus *	SRP039527
Eupolyphaga sinensis *	SRP039486
Periplaneta americana *	SRP039487
Anoplophora glabripennis	GALX00000000.1
Antheraea assama	GBZC00000000.1
Anthonomus grandis	GABY00000000.1
Bactrocera dorsalis	GAKP00000000.1
Belgica antarctica	GAAK00000000.1
Brassicogethes aeneus	GAPE00000000.1
Chrysopa pallens	GAGF00000000.1
Colaphellus bowringi	GBHN00000000.1
Corydalinae sp.	GADH00000000.1
Crioscolia alcione	GAXT00000000.1
Culicoides sp.	GAWM00000000.1
Dastarcus helophoroides	GBCX00000000.1
Delia antiqua	GAWI00000000.1
Fopius arisanus	GBYB00000000.1
Hypothenemus hampei	GDLS00000000.1
Ips typographus	GACR00000000.1
Lymantria dispar	GDCN00000000.1
Musca domestica	GDAV00000000.1
Nevrorthus apatelios	GACU00000000.1
Nicrophorus vespilloides	GDKQ00000000.1
Oropsylla silantiewi	GAWY00000000.1
Osmia cornuta	GAGH00000000.1
Polistes metricus	GDHQ00000000.1
Raphidia ariadne	GACX00000000.1
Rhodinia newara	GBZE00000000.1
Samia ricini	GBZD00000000.1
Sitodiplosis mosellana	GAKJ00000000.1
Stomoxys calcitrans	GDIM00000000.1
Telchin licus	GBAS00000000.1
Telenomus podisi	GBEU00000000.1
Teleopsis whitei	GBBQ00000000.1
Tetramorium bicarinatum	GASM00000000.1
Thaumetopoea pityocampa	GBZB00000000.1
Themira biloba	GBGG00000000.1
Xyela alpigena	GADA00000000.1
Yponomeuta evonymellus	GASG00000000.1

*** represents newly sequenced transcriptomes.**

Table S3. Accession number of the 17 genomes of Anophelinae.

Species	Accession
Anopheles stephensi	ALPR00000000
Anopheles maculatus	AXCL00000000
Anopheles minimus	APHL00000000
Anopheles funestus	APCI00000000
Anopheles culicifacies	AXCM00000000
Anopheles merus	AXCQ00000000
Anopheles arabiensis	APCN00000000
Anopheles quadriannulatus	APCH00000000
Anopheles gambiae	AAAB00000000
Anopheles melas	AXCO00000000
Anopheles christyi	APCM00000000
Anopheles epiroticus	APCJ00000000
Anopheles farauti	JXWZ00000000
Anopheles dirus	APCL00000000
Anopheles sinensis	ATLV00000000
Anopheles atroparvus	AXCP00000000
Anopheles albimanus	APCK00000000
